# Endurance Sport and “Cardiac Injury”: A Prospective Study of Recreational Ironman Athletes

**DOI:** 10.3390/ijerph110909082

**Published:** 2014-09-03

**Authors:** Roman Leischik, Norman Spelsberg

**Affiliations:** School of Medicine, Faculty of Health, Witten/Herdecke University, Elberfelder Str. 1, 58095 Hagen, Germany; E-Mail: norman_spelsberg@yahoo.de

**Keywords:** endurance sport, cardiac injury, triathlon, marathon

## Abstract

*Background*: Participation in triathlon competitions has increased in recent years. Many studies have described left or right ventricular injury in endurance athletes. The goal of this study was to examine the right and left ventricular cardiac structures and function and dynamic cardio-pulmonary performance in a large cohort of middle- and long-distance triathletes. *Methods*: 87 triathletes (54 male and 33 female) were examined using spiroergometry and echocardiography. The inclusion criterion was participation in at least one middle- or long distance triathlon. *Results*: Male triathletes showed a maximum oxygen absorption of 58.1 ± 8.6 mL/min/kg (female triathletes 52.8 ± 5.7 mL/min/kg), maximum ergometer performance of 347.8 ± 49.9 W (female triathletes 264.5 ± 26.1 W). Left ventricular ejection fraction (EF) was normal (male triathletes EF: 61.9% ± 3%, female triathletes EF: 63.0% ± 2.7%) and systolic right ventricular area change fraction (RV AFC%) showed normal values (males RV AFC%: 33.5% ± 2.2%, females 32.2% ± 2.8%). Doppler indices of diastolic function were normal in both groups. With respect to the echocardiographic readings the left ventricular mass for males and females were 217.7 ± 41.6 g and 145.9 ± 31.3 g, respectively. The relative wall thickness for males was 0.50 ± 0.07, whereas it was 0.47 ± 0.09 for females. The probability of left ventricular mass >220 g increased with higher blood pressure during exercise (OR: 1.027, CI 1.002–1.052, *p* = 0.034) or with higher training volume (OR: 1.23, CI 1.04–1.47, *p* = 0.019). *Conclusions*: Right or left ventricular dysfunction could not be found, although the maximal participation in triathlon competitions was 29 years. A left ventricular mass >220 g is more likely to occur with higher arterial pressure during exercise and with a higher training volume.

## 1. Introduction

The possibility of myocardial damage due to physical activity has been known since ancient times. According to legend, a soldier named Pheidippides (more likely Philippides) dropped dead after running to Athens from the battle at Marathon with the news of victory [1]. A soldier or courier, he is purported to have already run 240 km from Athens to Sparta and back before running a marathon distance to Athens, and the truth about his story is unknown [2]. What is clear, however, is that this death is the first recorded sport-related death.

**Figure 1:**
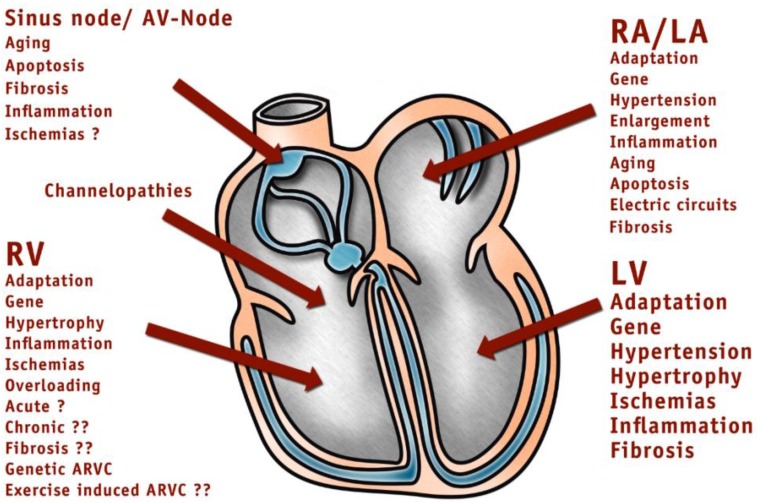
Factors that might influence heart function during endurance sport.

Some papers have reported on common deaths in triathlon competitions [3] and exercise-induced cardiac fatigue in triathlon competitions [4,5] and other endurance disciplines [6,7]. The problem of cardiac injury with endurance sports is a complex issue [7,8,9]. Figure 1 shows some factors that might influence cardiac injury in endurance athletes.

Triathlon is an endurance sport, especially when performed in middle- and long-distance formats (e.g., middle/long distance Ironman: a 1.9/3.8 km swim, a 90/180 km bicycle ride and a 21.1/42.2 km run), mainly under aerobic conditions. It is particularly important for triathletes to perform at sub-maximal levels over a long period to avoid reaching a state of exhaustion [10,11]. Amateur athletes typically take 5 to 6.5 h for middle-distance events and 10 to 16 h for long-distance events. Such long periods of stress require that both the amateur and the top athletes be adequately trained and have sufficient aerobic capacity [10]. A number of studies with small cohorts have reported spiroergometric data [12,13,14,15]. Problems regarding the condition of athletes’ hearts have been identified [16]. These problems are mainly known from observations of professional cyclists [17] and elite runners [18]. Regarding the literature about “exercise-induced” cardiac fatigue and “athlete’s heart”, the following questions have to be asked:

- Are there signs of cardiac “fatigue” when triathletes have trained hard over a period of years? Are there risks for right ventricular or left ventricular dysfunction for amateur athletes?
- What type of athletes’ hearts or what type of hypertrophy, concentric or eccentric, is found most often in triathletes?
- Are there signs of reduced physiological performance?

## 2. Experimental Section

### 2.1. Participants

Eighty-seven triathletes were examined, including 54 males and 33 females. All triathletes were examined by echocardiography (except three males) and spiroergometry. Triathletes underwent their annual medical check-up or examination for training preparation in 2011/2012, which would have been performed as part of the normal clinical routine. All examinations were performed by Dr. Roman Leischik or Dr. Norman Spelsberg. The participants were invited to participate in the study during their routine check-up or routine training preparation, and announcements in social media and triathlon clubs were also made. Dr. Roman Leischik is the medical supervisor of the Triathlon Club PV-Witten since 2007. This study was prospective in design.

### 2.2. Methods: Echocardiography and Spiroergometry

A Vivid 7 echocardiograph, produced by General Electric (Fairfield, CT, USA) was used for the examinations. The Ergobike 8I, produced by Daum (Fürth, Germany), and the Metalizer 3B, produced by Cortex (Leipzig, Germany), were used for the spiroergometric examination.

To compare the data from elite triathletes with those of non-elite triathletes, both males and females were divided into two groups according to their aerobic capacity (relative VO_2_/min/kg at the aerobic threshold). The 20 males and 15 females with the highest values were designated as elite.

All triathletes were examined in a single day using first echocardiography and then spiroergometry. All examinations were performed in 2011 and 2012 at the Sports Medicine Center, Hagen, Germany. The spiroergometry was performed in the following manner: the stress test was conducted in stages after successful gas and volume calibration at 50 W for 3 min, 100 W for an additional 3 min, after which it increased by another 30 W for 3 minutes. The test ended when the subject was exhausted.

The echocardiographic analysis was conducted according to general recommendations [19,20]. The formula recommended by the American Society of Echocardiography (ASE) was used to calculate the muscle mass. EDV and ESV were determined as monoplane by the modified Simpson method.

The spiroergometric analyses were conducted according to the literature. VAT was determined as the first non-linear increase in the ventilatory equivalent for oxygen without a simultaneous increase of the ventilatory equivalent for CO_2_. RCP was determined as a simultaneous non-linear increase of both ventilatory equivalents according to recommendations [21,22]. VO_2max_ was registered as the highest average value of oxygen absorption over 30 seconds. Elite triathletes were identified by their aerobic capacity. The 20 males and 15 females with the best values of relative oxygen uptake (in mL/min/kg) were classified as elite.

#### 2.2.1. Ethics Statement

All athletes gave verbal and written consent to voluntary performance testing and for use of their data in this study. All data were anonymized. Triathletes underwent their annual medical check-up or examination for training preparation in 2011/2012, which would have been performed in any case as it is clinical routine. The study was approved as a doctoral dissertation by the Dissertation Audit Committee of the University Witten/Herdecke. All examinations (echocardiography and spiroergometry) were a part of the routine clinical and diagnostic care for the triathletes. All examinations were performed by two experienced investigators (see chapter methods). This study did not introduce any pharmaceutical interventions or changes in the clinical course of the triathletes.

#### 2.2.2. Statistical Analyses

The entire statistical analysis was designed as follows. Stata/IC 11.2 for Windows was used for data preparation and statistical analysis. The Mann-Whitney U-Test was used to compare the groups. The Kaplan-Meier product limit method and the Cox proportional hazards model were used to estimate the odds ratios for analysis of the relationship between arterial pressure and the probability of a left ventricular muscle mass (LVM) >220 g. All statistical tests were two-sided, with a significance level of 0.05.

## 3. Results and Discussion

### 3.1. Participants Characteristics

Anthropometric data and the general cardiac parameters of the participants are listed in Table 1.

Male triathletes have significant greater weight, BMI, and BSA than female triathletes, but lower body fat (12% *vs*. 22.8%). Heart cavities and stroke volume are significant larger, without differences in systolic function (EF) or diastolic function (Doppler parameters).

**Table 1:** Anthropometric and structural echocardiographic characteristics of the study population.

Parameters	Male			Female			Mann-Whitney U-Test *p*-value
	n	Mv	Sd	n	Mv	Sd	
Age (years)	54	38.1	11.8	33	34.3	8.1	0.137
Weight (kg)	54	76.8	8.9	33	61.5	7.8	0.001
Size (cm)	54	182.4	6.7	33	168.8	6.4	0.001
BMI (kg/m²)	54	23.0	1.83	33	21.6	2.28	0.001
BSA (m²)	54	1.97	0.14	33	1.70	0.13	0.001
%body fat	54	12.5	3.6	33	22.8	4.7	0.001
Ao	51	2.90	0.37	33	2.47	0.24	0.001
LA	51	2.54	0.28	33	2.35	0.25	0.002
LAV (mL)	51	29.1	7.8	33	27.4	9.3	0.254
IVS thickness diastolic (cm)	51	1.23	0.13	33	1.02	0.17	0.001
IVS thickness systolic (cm)	51	1.67	0.18	33	1.44	0.22	0.001
PWT diastolic (cm)	51	1.22	0.14	33	1.02	0.16	0.001
PWTs (cm)	51	1.70	0.17	33	1.48	0.20	0.001
Relative wall thickness	51	0.50	0.07	33	0.47	0.09	0.066
LVEDD (cm)	51	4.8	0.38	33	4.4	0.32	0.001
LVESD (cm)	51	3.3	0.29	33	2.9	0.27	0.001
LVM (g)	51	217.7	41.6	33	145.9	31.3	0.001
LVM (g/m²)	51	110.5	21.8	33	85.8	18.7	0.001
LVEDV (mL)	51	138.5	22.3	33	105.0	17.8	0.001
LVESV (mL)	51	52.7	9.9	33	38.9	7.1	0.001
SV (mL)	51	85.7	14.0	33	66.1	11.3	0.001
EF (%)	51	61.9	3.0	33	63.0	2.7	0.292
LVOT V_Max_ (m/s)	51	0.80	0.13	32	0.86	0.13	0.047
MV E_Max_ (m/s)	51	0.53	0.10	33	0.56	0.12	0.091
MV A_Max_ (m/s)	51	0.36	0.06	33	0.38	0.09	0.569
MV E/A Ratio	51	1.48	0.31	33	1.54	0.34	0.407
RV parasternal	51	3.18	0.13	33	2.40	0.18	0.001
RV AFC%	51	33.5	2.2	33	32.2	2.8	0.005

n = number; Mv = mean value; Sd = standard deviation; BMI = body mass index; BSA = body surface area; Ao = aortic diameter; LA = left atrial diameter in cm; LAV = left atrial end-systolic volume; IVS = interventricular septum; PWT = diastolic posterior wall thickness; PWTs = systolic posterior wall thickness; RWT = relative wall thickness (formula: 2 × PWT/LVEDD); LVEDD = left ventricular end-diastolic diameter; LVESD = left ventricular end-systolic diameter; LVM = left ventricular mass; LVM (g/m^2^ ) = LVM/BSA; LVEDV = left ventricular end-diastolic volume; LVESV = left ventricular end-systolic volume; d = diastolic; s = systolic; SV = stroke volume; EF = left ventricular ejection fraction in %; LVOT V_Max_ = left ventricular outflow tract velocity; MV E_Max_ = early (E) mitral velocity; MV A_Max_ = mitral A (atrial) velocity; RV parasternal = RV diameter from parasternal view in cm; RV AFC = right ventricular area fractional change in %.

#### 3.1.1. Echocardiography

Myocardial hypertrophy was common and was classified according to Lang *et al*. [19] (Table 2 and Table 3).

**Table 2:** Myocardial hypertrophy in male triathletes.

Left Ventricle	Normal	Light	Moderate	Strong
LVEDD	51 (100%)	0	0	0
IVSD	3 (5.9%)	39 (76.5%)	9 (17.6%)	0
PWT	4 (7.8%)	37 (72.5%)	10 (19.7%)	0
LVM (g/m²)	31 (60.8%)	11 (21.6%)	7 (13.7%)	2 (3.9%)
LVEDV *	44 (86.3%)	4 (7.8%)	2 (3.9%)	1 (2.0%)

* Reference values according to Lang *et al*. [19]; LVEDD: left ventricular end-diastolic diameter; IVSD: interventricular septum diastolic thickness; PWT: diastolic posterior wall thickness; LVM (g/m²): Left ventricular mass/BSA; LVEDV: left ventricular end-diastolic volume.

**Table 3:** Myocardial hypertrophy in female triathletes.

Left Ventricle	Normal	Light	Moderate	Strong
LVEDD	33 (100%)	0	0	0
IVSD	12 (36.4%)	18 (54.5%)	3 (9.1%)	0
PWT	12 (36.4%)	18 (54.5%)	3 (9.1%)	0
LVM (g/m²)	26 (78.8%)	4 (12.1%)	1 (3.0%)	2 (6.1%)
LVEDV *	18 (54.5%)	5 (15.2%)	8 (24.2%)	2 (6.1%)

* Reference values according to Lang *et al*. [19]; LVEDD: left ventricular end-diastolic diameter; IVSD: interventricular septum-end-diastolic thickness; PWT: diastolic posterior wall thickness; LVM (g/m²): Left ventricular mass/BSA; LVEDV: left ventricular end-diastolic volume.

In the study population, concentric changes of the left ventricle characterized the echocardiographic morphological picture. Concentric remodeling for males was found in 26 cases and concentric hypertrophy was observed in 21 cases. One male triathlete had eccentric hypertrophy, and three had normal myocardial anatomy. Seventeen females displayed concentric remodeling, and 6 females had concentric hypertrophy. Cardiac adaptation forms are visualized in Table 4.

**Table 4:** Cardiac adaptation in the study collective *.

**RWT >0.42 cm**	**Concentric Remodeling**	**Concentric Hypertrophy**
	Males: 26	Males: 21
	Females: 17	Females: 6
**RWT <0.42 cm**	**Normal**	**Eccentric Hypertrophy**
	Males: 3	Males: 1
	Females: 9	Females: 1

* Reference values and definition according to Lang *et al*. [19]; RWT: relative wall thickness (formula: 2 × PWT/LVEDD).

The classification into types of hypertrophy followed the criteria of Lang *et al*. [19]. Right ventricular remodeling or other pathological findings in the right ventricle were not found. Left ventricular function was excellent in all triathletes, even if they trained hard and had finished middle- or long-distance triathlons.

#### 3.1.2. Spiroergometry/Physiological Performance

Oxygen absorption, ergometer performance and heart rate with VAT, RCP and at peak capacity are shown in Table 5 (males) and Table 6 (females).

**Table 5:** Males: heart rate, oxygen uptake and power output according to performance.

Measurements	Elite Males			Non-Elite Males			*p*-Value *
	n	Mv	Sd	n	Mv	Sd	
AT (aerobic threshold)							
HR	20	160.4	9.1	34	144.9	13.0	0.000
aVO_2_	20	3.85	0.46	34	3.14	0.52	0.000
rVO_2_	20	53.1	5.2	34	39.8	5.5	0.000
%VO_2max_	20	82.4	6.6	34	73.9	11.7	0.004
Watt	20	314.5	47.0	34	259.7	44.1	0.000
RCP (respiratory compensatory point, anaerobic threshold)							
HR	20	170.8	7.4	34	157.7	12.2	0.000
aVO_2_	20	4.37	0.61	34	3.59	0.48	0.000
rVO_2_	20	60.1	6.6	34	45.4	5.1	0.000
%VO_2max_	20	92.3	6.9	34	84.4	10.4	0.003
Watt	20	346.3	48.6	34	289.7	38.2	0.000
Peak capacity							
HR	20	180.4	7.1	34	144.9	13.0	0.000
aVO_2max_	20	4.7	0.7	34	4.3	0.6	0.060
rVO_2max_	20	64.7	6.7	34	54.2	7.1	0.000
Watt_max_	20	367.0	47.6	34	336.5	48.3	0.026

n = number; Mv = mean value; Sd = standard deviation; aVO_2_ = absolute VO_2max_ in L/min; rVO_2_ = relative VO_2_ in mL/kg^−1^·min^−1^; * = of the Mann-Whitney U-Test; HR = heart rate; Watt = power output.

**Table 6:** Females: heart rate, oxygen uptake and power output according to performance.

Measurements	Elite Females			Non-Elite Females			*p*-Value *
	n	Mv	Sd	n	Mv	Sd	
VAT (ventilatory aerobic threshold)							
HR	15	160.6	14.5	18	138.6	19.9	0.002
aVO_2_	15	2.76	0.35	18	1.99	0.46	0.001
rVO_2_	15	45.5	5.4	18	32.7	3.8	0.001
%VO_2_	15	81.2	7.0	18	64.4	12.9	0.001
Watt	15	230.0	24.5	18	162.2	41.4	0.001
RCP (respiratory compensation point, anaerobic threshold)							
HR	15	169.8	12.6	18	152.3	21.0	0.016
aVO_2_	15	3.06	0.28	18	2.33	0.56	0.001
rVO_2_	15	46.8	14.9	18	37.4	6.3	0.001
%VO_2max_	15	91.7	6.7	18	75.3	14.7	0.001
Watt	15	252.5	23.8	18	195.0	44.0	0.001
Peak capacity							
HR	15	179.7	10.0	18	179.8	7.6	0.971
aVO_2max_	15	3.4	0.3	18	3.1	0.3	0.006
rVO_2max_	15	56.1	5.7	18	50.1	4.1	0.004
Watt_max_	15	274.0	32.5	18	256.7	16.4	0.097

n = number; Mv = mean value; Sd = standard deviation; aVO_2_ = absolute oxygen uptake in L/min; rVO_2_ = relative oxygen uptake in mL/kg^−1^·min^-1^; * = Mann-Whitney U-Test; HR = heart rate; Watt = power output; max = maximum.

#### 3.1.3. Left Ventricular Hypertrophy

According to the values reported by Devereux *et al*. [23] and Bove *et al*. [24], we divided male triathletes into two groups, group 1 with LVM >220 g and group 2 with LVM <220 g, to assess the possible reasons for left ventricular hypertrophy. The significant differences between the two groups are visible in Table 7.

**Table 7:** Differences between males with LV-mass >220 g and <220 g.

LV Mass (ASE) g	Male < 220 g			Males > 220 g			*p*-Value
	n	Mv	Sd	n	Mv	Sd	Mann-Whitney U-Test
VO_2AT_	27	3.2	0.5	24	3.7	0.5	0.001
Training distance bike	27	190.3	65.8	24	250.2	60	0.004
SV (Teich) mL	27	63	11.7	24	72.9	13.3	0.006
SV index (Teich) mL/m²	27	32.2	5.9	23	36	6.3	0.018
Left atrium (cm)	27	2.46	0.27	24	2.64	0.27	0.020
Watts_AT_	27	265.6	46.6	24	301.3	53.4	0.023
Training time bike	27	7	2.2	24	8.6	2.4	0.034
Training time (overall)	27	15.7	2.7	24	17.8	3.3	0.035
Watt_max_	27	336.7	41.9	24	363.8	56.6	0.042
Training time swimming	27	3.2	1.2	24	3.8	1.4	0.049
RRsWatt_max_	27	188.1	20.4	24	199.6	19.9	0.055
RRs_AT_	27	178	24.6	24	192.9	20.5	0.056
VO_2max_	27	4.3	0.5	24	4.6	0.8	0.059

VO_2AT_: oxygen uptake at the aerobic threshold (VAT) in L/min; Training-distance bike: kilometers/week; SV (Teichholz) mL: left ventricular stroke volume; Training time bike: hours/week Training time overall: hours/week Watt_max_: maximal power; Training time swimming: hours/week; RRs_Wattmax_: systolic arterial pressure during maximal exercise; RRs_AT_: systolic arterial pressure at the aerobic threshold; VO_2max_: maximal oxygen uptake in L/min.

Additional results about the possible reasons for LV hypertrophy were calculated by odds ratios (Table 8).

**Table 8:** Probability of LVM >220 g: odds ratios.

Parameters	LVM				OR	95%-CI		*p*-Value
	<220 g (*n* = 27)		>220 g (*n* = 24)					
	Mv	SD	Mv	SD				
BPsRest	125.4	10.8	130.8	15.7	1.031	0.989	1.074	0.148
BPs Aero.thresh.	178.0	24.6	192.9	20.5	1.027	1.003	1.051	0.025
BPs Anae.thresh.	185.2	21.5	198.8	22.3	**1.027**	**1.002**	**1.052**	**0.034**
BPsW_max_	188.1	20.4	199.6	19.9	1.027	1.000	1.054	0.050
Tr.time swim	3.2	1.2	3.8	1.4	1.46	0.95	2.25	0.081
Tr.time bike	7.0	2.2	8.6	2.4	1.33	1.06	1.66	0.015
Tr.time run	4.9	1.5	4.9	1.2	1.05	0.70	1.56	0.823
Training time	15.7	2.7	17.8	3.3	**1.23**	**1.04**	**1.47**	**0.019**
Triathlon since y	14.5	9.0	15.7	10.3	1.01	0.96	1.07	0.654

BPs = systolic blood pressure (mmHg); Tr.time = training time in hours/week; Aero.thresh. = aerobic threshold; Anae.thresh. = anaerobic threshold; y = years.

With respect to the relationship between arterial hypertension and myocardial hypertrophy, 32 male triathletes had a wall thickness >1.2 cm, whereas 19 had a thickness <1.2 cm. In these male athletes, the mean arterial blood pressure values of 196.9 mmHg and 187.9 mmHg (*p* = 0.075) were measured at the maximal watts level (W_max_). The odds ratios showed a significant relationship between the arterial pressure values during exercise (at the aerobic and anaerobic threshold). There was an additional relationship between the overall training time and a LVM >220 g (Table 8).

#### 3.1.4. Training Habits/History

The mean training times were similar in male and female triathletes (Table 9). The mean time for swimming was 3.5 h/week, for biking 6–8 h a week, for running 5 h a week. Total training time was 14–15 h/week. The maximal training time for triathlon was 29 years for a 64 years old male triathlete. The oldest female triathlete was 45 years old and had trained for triathlons for 5 years. Four athletes had a history of *Chlamydia pneumoniae* infections.

**Table 9:** Training habits/week.

Trainings	Males			Females			Times/distances
	n	Mv	Sd	n	Mv	Sd	
Swimming	54	3.5	1.28	33	3.6	1.19	hours
	54	7.8	3.9	33	7.5	3.5	km
Biking	54	7.8	2.39	33	6.6	2.05	hours
	54	218.6	68.9	33	168.5	70.9	km
Running	54	4.9	1.36	33	5.1	1.36	hours
	54	52.4	13.3	33	51.5	14	km
Tr.time/week	54	16.7	3.1	33	15.5	3.3	hours
Triathlon years	54	9.0	6.4	33	6.4	4.5	years
Sport years	54	14.9	9.4	33	13.7	6.8	years

Mv = mean value; Sd = standard deviation.

### 3.2. Discussion

Sports-related cardiac injury is a very broadly discussed issue [7,8,9]. The most substantial cardiac injury is sport-related sudden death. Most cases of sudden death in athletes over 35 years of age are caused by coronary disease [25], and their prognoses depend on the coronary arteriosclerosis burden [26] rather than on a possible increase in biomarkers during competition. The sudden deaths of young athletes <35 years (Table 10) typically have different causes (hypertrophic cardiomyopathy, coronary disease, coronary anomalies, myocarditis and arrhythmogenic right ventricle) [27,28,29,30].

In this study, pathological structural cardiac changes could not be observed. The dynamic physiological performance of the triathletes showed high baseline values without functional signs of cardio-pulmonary impairment.

**Table 10:** Causes of sudden cardiac death in young athletes 12–35 years old (percentages).

Causes of cardiac sudden death	Maron [27]	Corrado [29]	Solberg [30]	Marijon [28]
Aortic rupture	2.2	1.8	4.3	2
Aortic stenosis/cong. HD	1.8		4.3	6
ARVC	4	22		4
Channelopathies (QT, WPW)	3	1.8	8.7	12
Coronary artery anomalies	24	11	3.3	
Coronary disease	3	18	48	6
Dilatative CM	2	1.8		4
Hypertrophic CM	36	1.8	4.3	10
MVP	4	7.3		2
Myocarditis	5.4	9	22	4
Possible HCM	11.3			4
Riva muscle bridge	2.2	3.6		2
Unclear		1.8		36
	*n* = 1049	*n* = 55	*n* = 22	*n* = 50

cong. HD = congenital heart disease; ARVC = arrhythmogenic right ventricular cardiomyopathy; QT = QT-syndrome (including Romano-Ward syndrome and Jervell-Lange-Nielsen syndrome); WPW = Wolff-Parkinson-White syndrome; CM = cardiomyopathy; HCM = hypertrophic cardiomyopathy; MVP = mitral valve prolapsed.

#### 3.2.1. Cardiac Adaptation to Exercise and Left Ventricular Hypertrophy

The specific endurance training of triathletes leads to physiological changes in their performance parameters [31] and results in changes in cardiac function or cardiac structures [32]. This adaptation is linked to the nature and magnitude of the physical exercise [16,33]. The physiological adaptation is a “harmonic increase in [the] size” of a healthy heart caused by physical activity [34]. The term “athlete’s heart” [16] has been used since 1899 [35]. The sudden death of athletes is more often a problem for male athletes [28]. Different causes of sudden death [27], (silent coronary disease [30], hypertrophic cardiomyopathy [27] and arrhythmogenic right ventricular cardiomyopathy [29]) have been reported. Cardiovascular adaptations to exercise have been systematically defined and differ according to the type of conditioning endurance training [33,36,37]. Cycling and rowing have the biggest effects on the cavity size and wall thickness [27,38].

Concentric hypertrophy in triathletes has already been described [39]. Douglas *et al*. [40] suggested that athletes developed hypertrophy from the systolic blood pressure increase during exercise, which could be explained by the frequency of the training. The diastolic function was normal under these conditions.

In the present study, odds ratio analysis showed a relationship between myocardial thickening and exercise arterial pressure. Intense, long-term training with exercise-induced blood pressure elevation can lead to hypertrophy that mimics the conditions of pathological arterial pressure changes. The male triathletes with a wall thickness >1.2 cm had higher arterial pressure (196.9 mmHg *vs*. 187.9 mmHg; *p* = 0.075) at the peak capacity (maximum wattage), but this difference was not significant. This difference was found only in males; the average arterial pressure values for females were not different and did not increase. Indicators of an increase in wall thickness additional other than dilatation have been confirmed in a study of female athletes [41] that compared long-distance and short-distance runners. Endurance training for females causes an increase in the wall thickness rather than the dilatation of the left ventricle. However, the RWT reported in that study is significantly lower than what we observed (0.384 ± 0.052). The basic assumption for triathletes that this sport can lead to eccentric rather than to concentric hypertrophy was not confirmed in this study. There were signs of remodeling with primarily concentric hypertrophy in the female subjects in the present study. Concentric remodeling for males was found in 26 cases, and concentric hypertrophy could be observed in 21 cases. Concentric remodeling and concentric hypertrophy are more common in male athletes [16]. Different authors have concluded that strength training predominantly leads to concentric hypertrophy and endurance training leads to eccentric hypertrophy [42]. In this study, concentric remodeling was the most frequently observed. George *et al*. [16] reported that the expected pattern of eccentric enlargement was replaced by a pattern of concentric or symmetric enlargement in groups of highly trained athletes. Generally, the adaptation of the cardiac mass does not appear to depend on the type of conditioning [16]. In our opinion, the results of wall thickness measurements depend on the cohort size, age and gender.

In 1989, Douglas *et al*. [43] published a comparison of 36 triathletes comprising 17 normal control and 15 high-pressure subjects. The authors reported that the triathletes underwent cardiac adaptations similar to pressure overload of the left ventricle and described an RWT of 0.41. In these triathletes, the left ventricular muscle mass was positively correlated with the systolic blood pressure during exercise.

The difference between triathletes and cycle racers is that the training not only takes place under strength/endurance conditions, as during a cycle race, but it also occurs under running conditions. Modified strength training also results in different changes in the cardiac structures of triathletes compared to professional cycle racers [44]. In a study of 14 professional cycle racers, the authors found that the left ventricular muscle mass as well as the diastolic cardiac parameters resulted in eccentric hypertrophy more than concentric hypertrophy, which contrasts with our study. The functional changes of the cardiac structures for triathletes resemble the changes in runners [45].

#### 3.2.2. Right or Left Ventricular “Fatigue”

Some papers have reported that excessive endurance training may cause negative remodeling of cardiac structures [9,46,47]. Predominantly, ultra-marathons and Ironman-distance triathlons can cause transient overload of the right ventricle [46,47,48]. Fibrosis of the left ventricle in older runners is a possible cause of death [49,50]. Diverse patterns of myocardial fibrosis were reported by Wilson *et al*. [50]. Heidbüchel [51] postulates that load-induced, right ventricular arrhythmogenic cardiomyopathy could lead from repetitive microtrauma to chronic and structural changes of the right ventricle and “pro-arrhythmogenesis”. In his view, ventricular tachycardia originating from the right ventricle is responsible for the acute deaths. His hypothesis is based on a retrospective analysis of electrophysiological examinations in 2003 [52].

The discussion concerning cardiac injury and endurance sports is ongoing [7,8,9,53]. Pelliccia *et al*. [54] examined the left ventricular function of 114 Olympic athletes in a long-term follow-up study. The global left systolic function was unchanged, and wall motion abnormalities were absent. D’Andrea *et al*. [55] examined the right ventricle function in 650 top-level athletes and found enlarged right ventricular and right atrial dimensions; they described this as a “physiologic phenomenon.” Additionally, long-term-training marathon runners did not suffer any long term injury of the right ventricle (including examination by advanced strain technology) [56].

The systolic function of the left ventricle was not negatively influenced in our study cohort. The right ventricular fractional area change was normal in all triathletes. The oldest triathlete in our study had competed in triathlons for 29 years. The diastolic function in our cohort was not materially impaired. The E/A ratio, however, was slightly lower, as reported by Douglas *et al*. [40]. The impact of exercise-induced arterial hypertension (EIAH) is known in healthy subjects [57]. There is strong evidence that athletes have a higher incidence of atrial fibrillation [58,59] and bradyarrhythmias with increasing age [58,60]. We do not know the definitive reasons for this (increased atrial pressure?), but EIAH and LVM can cause atrial fibrillation in endurance athletes. The general prevalence or incidence of EIAH in athletes is unknown. The problem of EIAH seems to occur more in male competitive athletes who undergo a vigorous training schedule [57]. Low-intensity training and aerobic exercise have been shown to help decrease blood pressure [61,62]. The hypertensive or non-hypertensive response to exercise seems to be related to hereditary factors [63], to aging or to the individual arterial stiffness [64]. It is crucial to define the people who are at risk and to start therapy if possible [65]. In our daily practice, we treat subjects with low-dose ACE inhibitors or AT1-blockers before training or competition. The dosage should be tested using an exercise test. The present study shows the relationship between EIAH and cardiac hypertrophy (Table 5).

#### 3.2.3. Dynamic Physiological Performance

A prerequisite of successful participation in middle- and long-distance triathlons is sufficient aerobic capacity [31]. It also appears that this is essential for avoiding possible damage due to long-term endurance training [4]. In the present study, 54 male athletes had a VO_2max_ of 58.1 mL/kg^−1^·min^−1^ and a VAT of 44.7 mL/kg^−1^·min^−1^. These data are in agreement with published data from smaller studies. The VO_2_ for the VAT was between 34 and 49 mL/kg^−1^·min^−1^ body weight in previously published studies, which depends on the subject group. The maximum oxygen absorption in the previously published studies had a range of 43 to 74 mL/kg^−1^·min^−1^ body weight among the different groups that were examined. In contrast, studies of groups with, for example, five world-class athletes [66] report higher results. However, it can be postulated that a VO_2max_ >55 mL/kg^−1^·min^−1^ body weight is a good marker for successful participation in an Ironman competition at both middle and long distances. The oxygen absorption at the aerobic threshold should be >45 mL/min/kg body weight for males and >35 mL/kg^−1^·min^−1^ body weight for females. In the present study, the examinations always took place using egometric peak capacity by bicycle; treadmill data can differ depending on the athletes’ background [13]. Bunc *et al*. [67] propose the use of VO_2max_ values for young Olympic triathletes, with males having an average of >65 mL/min/kg and females >60 mL/min/kg. This pertains to elite triathletes. In smaller studies using bicycle ergometry, the maximum oxygen absorption values for female athletes (e.g., [68]) averaged 57.5 mL/kg^−1^·min^−1^ body weight, and the average oxygen absorption value for ten Olympic-level female athletes [69] was at VAT of 37.7 mL/min/kg body weight. The overall male and female group examined here had a physiological profile that matches well-trained and ambitious triathletes, though it does not compare to the equivalent values for world-class athletes.

When comparing the performance values of elite and non-elite male athletes (Table 2) in the present study, a significant difference was observed, as expected (relVO_2max_ for males 64 ± 6.7 *vs*. 54.9 ± 6.7 mL/kg^−1^·min^−1^ and VO_2_ for VAT 53.1 ± *vs*. 39.8 ± 5.5 mL/kg^−1^·min^−1^). A similar difference in the range of VO_2max_ was observed for female athletes (Table 3). In summary, the results from this study show a difference in VO_2max_, which is not as high as observed in triathletes examined in other studies. The values determined for German elite and amateur athletes are within the range of internationally determined parameters.

#### 3.2.4. Study Limitations and Future Directions

The cross-sectional design of this study did not allow us to exclude the possibility of negative effects of endurance sport in the triathletes, although our data suggest that our examined triathletes had no significant signs of cardiac injury except for the signs of the hypertrophy. The pathological relevance of the athletes’ hypertrophy is under discussion. Confirmatory longitudinal studies are necessary. It is not possible to exclude, that using of strain or tissue Doppler technique might provide diagnosis of abnormal cardiac function. Biplane analysis of the ventricular volumes might provide small changes in the stroke volume analysis, but in participants without regional wall motion abnormalities or cardiac infarction they are negligible.

## 4. Conclusions

The data of the present study on German triathletes participating in the middle- and long-distance events allow for the practical use of this information as a routine part of sports medicine in classifying the efficiency of performance diagnostic centers. In males, the training goals aim for an aerobic capacity of VAT = 45 mL/kg^−1^·min^−1^, (males) or 35 mL/kg^−1^·min^−1^ (females). In terms of the echocardiographic measurement, we can expect concentric remodeling/concentric hypertrophy. Valve dysfunctions are possible but rare. Accordingly, the blood pressure values should be thoroughly examined during routine or pre-event check-up. We conclude that the relationship between myocardial thickening and arterial blood pressure during exercise remains an open issue. With respect to echocardiography, we did not observe cardiac dysfunction in our athletes.

The idea of exercise-induced cardiac disease was suggested by Heidbüchel [51] and LaGerche [46] but the data are controversial [8,53]. In our cohort, with the mean time of participation in triathlon competitions of 9.0 years in males and 6.4 years in females (Table 9, we did not find the negative long-term effects on cardiac structures that had been proposed for exercised-induced cardiac fatigue. The longest time for participating in triathlon competitions was 29 years (one 64-year-old triathlete, with Ironman-distance times of *ca.* 11 h).

There is no doubt that athletes have a higher incidence of atrial fibrillation [58] and bradyarrhythmias [60]. Elite athletes (particularly men) have improved longevity because of the rare occurrence of cardiovascular disease [70]. Early death in individuals due by myocardial fibrosis is possible [49]. The general incidence of exercise-induced cardiac injury is not known, and the dose of exercise bouts and individual sensitivity has yet to be defined and should be evaluated in further prospective studies [71]. In all athletes with suspicious inflammation/myocarditis or in cases of power/performance lost, blood tests must be performed, especially for *Chlamydia pneumoniae* [72] or other bacterial/viral infections. The impact of exercise-induced arterial hypertension in endurance athletes remains an open issue [57]. Abnormal exercise-induced cardiac hypertrophy might be a risk factor for arrhythmia [57]. Further prospective studies on possible cardiac “negative remodeling” due to participation in sports (exercise-induced cardiac fatigue) with larger cohorts and under clearly defined conditions should be conducted. In addition, the optimal training volume of physical activity concerning the general survival rate should be investigated prospectively. Considering the dominant probability of coronary heart disease in ambitious athletes >35 years, risk stratification with exercise-tests/imaging techniques [73,74] is advisable in addition to basic examination (medical history/physical examination/12-channel-ECG). Marijon *et al*. [28] reported a five-fold higher cardiac mortality in young, ambitious competitive athletes (relative risk 9.8, 95%, CI 3.7 to 16) than in non-competitive athletes (2.2, 95%, CI 1.4 to 3.0). This fact supports the need for a more detailed examination of athletes <35 years and to identify people who are at risk.

Given the value of competitive sporting activities to hobby-athletes, the media, and other industries, physical activity in the general population is of fundamental importance [75]. Further prospective examinations and studies about the long-term adverse effects of endurance sports should be performed.
